# Determinants of new wavefront locations in cholinergic atrial fibrillation

**DOI:** 10.1093/europace/euy235

**Published:** 2018-11-23

**Authors:** Caroline H Roney, Fu Siong Ng, Michael T Debney, Christian Eichhorn, Arun Nachiappan, Rasheda A Chowdhury, Norman A Qureshi, Chris D Cantwell, Jennifer H Tweedy, Steven A Niederer, Nicholas S Peters, Edward J Vigmond

**Affiliations:** 1 ElectroCardioMaths Programme, Imperial Centre for Cardiac Engineering, Imperial College London, London, UK; 2 LIRYC Electrophysiology and Heart Modeling Institute, Bordeaux Fondation, Avenue du Haut-Lévèque, Pessac, France; 3 School of Biomedical Engineering and Imaging Sciences, King's College London, London, UK; 4 Univ. Bordeaux, IMB UMR 5251, F-33400 Talence, France

**Keywords:** Atrial fibrillation, Acetylcholine-induced atrial fibrillation, Wavefront dynamics, Dominant frequency, Phase singularity, Driver, Atrial Fibrosis

## Abstract

**Aims:**

Atrial fibrillation (AF) wavefront dynamics are complex and difficult to interpret, contributing to uncertainty about the mechanisms that maintain AF. We aimed to investigate the interplay between rotors, wavelets, and focal sources during fibrillation.

**Methods and results:**

Arrhythmia wavefront dynamics were analysed for four optically mapped canine cholinergic AF preparations. A bilayer computer model was tuned to experimental preparations, and varied to have (i) fibrosis in both layers or the epicardium only, (ii) different spatial acetylcholine distributions, (iii) different intrinsic action potential duration between layers, and (iv) varied interlayer connectivity. Phase singularities (PSs) were identified and tracked over time to identify rotational drivers. New focal wavefronts were identified using phase contours. Phase singularity density and new wavefront locations were calculated during AF. There was a single dominant mechanism for sustaining AF in each of the preparations, either a rotational driver or repetitive new focal wavefronts. High-density PS sites existed preferentially around the pulmonary vein junctions. Three of the four preparations exhibited stable preferential sites of new wavefronts. Computational simulations predict that only a small number of connections are functionally important in sustaining AF, with new wavefront locations determined by the interplay between fibrosis distribution, acetylcholine concentration, and heterogeneity in repolarization within layers.

**Conclusion:**

We were able to identify preferential sites of new wavefront initiation and rotational activity, in order to determine the mechanisms sustaining AF. Electrical measurements should be interpreted differently according to whether they are endocardial or epicardial recordings.

**What’s new?**

- We present a novel methodology to identify preferential sites of new wavefront initiation and rotational activity to determine the mechanisms sustaining atrial fibrillation (AF).
- Canine cholinergic AF is typically sustained by one dominant mechanism at any given time, either by rotational activity, or by new wavefronts/focal sources.
- Preferential phase singularity (PS) sites exist with high PS density around the left atrial/pulmonary vein junction.
- Canine cholinergic AF may exhibit preferential locations for new wavefront initiation.
- Computational simulations predict that only a small number of connection sites are functionally important in sustaining AF, with preferential new wavefront locations determined by the interplay between fibrosis distribution, acetylcholine concentration, and heterogeneity in repolarization.

## Introduction

Atrial fibrillation (AF) is supported by the electrical and structural substrates. Understanding how they interact may inform treatment strategies. There are multiple mechanisms proposed to underlie AF, including multiple wavelets,^1^^,^^2^ multiple foci,^3^ and rotational activity,^4^ and it can be difficult to distinguish between them using currently available clinical mapping technologies.

Clinically, spatio-temporal features of the electrical activity may be used to guide ablation therapy. For example, areas of high dominant frequency (DF) are targeted as they may coincide with electrical drivers; however, there is conflicting evidence regarding the stability of such sites.^5^^,^^6^ In addition, phase singularity (PS) analysis has been used in several clinical centres to indicate areas of rotational activity, and regions of high rotational content are ablated.^4^^,^^7^

Atrial fibrillation occurs on a three-dimensional substrate. The transmural dimension has been proposed as an important element in AF, with evidence for the existence of epicardial breakthrough^8^ and intramural rotors.^9^ The electrical activity on the endocardium and epicardium of the atrium during AF have been shown to exhibit degrees of discordance, in which there are periods where these two surfaces show the same wavefront pattern, and times when they have different wavefront patterns.^10^

Most mapping modalities are restricted to recording measurements from a single surface of the atrium only. Consequently, it may be difficult to differentiate between potential causes for focally propagating wavefronts, which include triggered activity, intramural re-entry, and breakthrough due to transmural conduction. In addition, during AF it is often difficult to distinguish continuous rotor or multiple wavelet activity from new wavefronts following electrical quiescence in the mapping field. These mechanisms may be visually indistinguishable; however, areas of new wavefronts may represent critical regions responsible for sustaining AF that are not detected by PS density maps or DF maps. Identification of foci responsible for sustaining AF may have clinical utility as potential ablation targets.

In this study, we investigate arrhythmia mechanisms, including detecting instances of new wavefronts and rotational activity, in order to identify drivers responsible for sustaining AF. We applied the method to a canine cholinergic AF preparation and a computational model of AF. We found that AF is typically sustained by one dominant mechanism at any given time, either by rotational activity, or by new wavefronts/focal sources. The computational model offered insight into the determinants of preferential sites of new wavefront initiations. Our novel method may be used to identify preferential sites of new wavefront initiation and rotational activity in order to determine the mechanisms sustaining AF.

## Methods

### Experimental preparation

All animal experiments complied with UK Home Office standard regulations as designated by the EU Directive 2010/63/EU. Four 18-month-old healthy Beagle dog hearts were kindly donated by GlaxoSmithKline, after the animals were necessarily sacrificed at the end of their pharmacology/toxicology studies. Only control animals were used in this study. The dogs were euthanized with an overdose of pentobarbital and the hearts removed immediately after confirmation of death. Hearts were immersed in 4°C cardioplegia solution (Plegivex, Ivex Pharmaceuticals, UK; in mM: NaCl 147; KCl 16; MgCl_2_ 16; CaCl_2_ 1.2; Procaine hydrochloride), and then transported to the laboratory (transport time ∼2 h). For each heart, a circumflex artery-perfused, isolated left atrial (LA) preparation was dissected as previously described,^11^^,^^12^ and perfused in a temperature controlled bath (37°) with Tyrode’s solution. A bipolar electrode was attached to the endocardial surface for monitoring the electrical activity and AF identification. The preparation was loaded with a voltage sensitive dye (∼60μL of 1 mg/mL RH237 in DMSO, Invitrogen, Paisley, UK) and perfused with 10 µmol/L blebbistatin (Sigma-Aldrich, Gillingham, UK) to eliminate motion artefact. Hearts were illuminated using monochromatic LEDs, and excited at wavelength 530 nm. Emitted light was focused, filtered (>630 nm), and detected using a CMOS (complementary metal oxide semiconductor) optical mapping camera with 128-by-80 pixels. A dynamic pacing protocol was applied to assess the action potential duration (APD) and conduction velocity (CV) restitution properties of the tissue, pacing from 4000 ms down to the refractory period. Acetylcholine (10–30μM) was added to the preparation and burst pacing was used to induce sustained AF. Multiple sequential ten second recordings of AF were then analysed.

### Signal filtering

All raw optical signals were processed in Matlab by applying a sequence of steps to reduce noise, using a modified version of the optical mapping toolbox from the Efimov laboratory.^13^ Briefly, signals were spatially filtered with a convolution bin size of 9-by-9 pixels; and temporally filtered using a finite impulse response zero-phase band-pass filter (Parks-McClellan algorithm; firpm, filtfilt) from 2 Hz to 125% of the DF. Finally, baseline drift was removed and normalization applied. The choice of spatial bin size and the high-band of the temporal filter were determined in initial parameter sensitivity testing.

### Phase calculation

Phase was calculated to assess the wavefront dynamics. To calculate phase angle using the Hilbert transform, a zero mean signal was required. As such, a pre-processing step was applied to the signals, similar to the pseudo-empirical mean technique of Bray and Wikswo.^14^ Maxima and minima in the signal were defined based on a sliding window of length equal to 90% of the average LA cycle length (CL), and pairs below an amplitude threshold were removed (15% of median amplitude). The maxima and minima were joined using cubic splines, respectively and used to normalize the signal. These parameters were found to give an optimal set of deflections a full trajectory in the phase plane and appropriate phase angle.

The phase angle was then spatially smoothed to further reduce noise content and to enable accurate identification of PSs. A 9-by-9 convolution operator was applied to the exponential form of the phase angle, to properly assign phase across the branch cut.

Phase singularities were identified by using the topological charge technique of Bray and Wikswo.^15^ Due to noise in phase, points were defined as candidate PSs if the integral was within 3.0 of ±2π, and an actual PS if at least three of the eight neighbouring pixels were also candidate PSs and if the pixel is the closest of them to ±2π.^16^ Phase singularities were then tracked over time, and those lasting for greater than one rotation were defined to be rotors. Dominant frequency maps and PS distribution maps were calculated.

### Detecting instances of new wavefronts

Instances of new wavefronts on the endocardium were identified as areas of active tissue where there were no active tissue pixels in the vicinity in the previous frame. This was achieved by defining active tissue pixels as those at an isophase value of −π/2. To calculate an acceptable isophase line without the need to interpolate the data, the method suggested by Kay and Gray^17^ was used. Pixels are defined as belonging to an isophase line of a given value θ if the pixel phase lies within the range [θ − 0.5, θ], and between one and three of its four adjacent neighbouring pixels have a value greater than θ.

**Figure 1:**
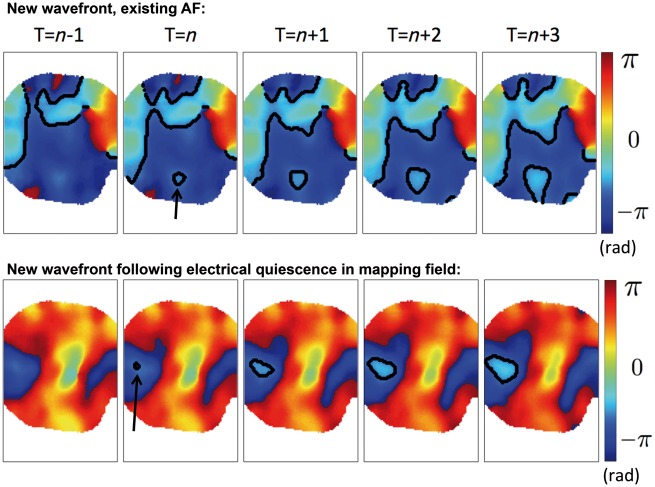
New wavefronts are identified as instances of active pixels that cannot be explained by propagation from active pixels in the previous frame, and occur during existing AF or following electrical quiescence in the mapping field. Sequential frames are shown in which a new wavefront in the mapping field is seen in frame *T* = *n*, which cannot be explained by any nearby active pixels in frame *T* = *n−1*. The new wavefront is seen to propagate focally. Top row: there are other wavefronts within the mapping field at *T* = *n-1*, and so this is termed to be a new wavefront, in which there is existing AF. Bottom row: there are no active pixels in frame *T* = *n−1*, and so this is termed to be a new wavefront following electrical quiescence in the mapping field. AF, atrial fibrillation.

For each wavefront within a given frame, there are three possibilities: the wavefront is an existing wavefront, present in the previous frame (*propagated wavefront*); the wavefront is a new wavefront and there are other wavefronts present within the mapping field (*new wavefront, existing AF*); the wavefront is a new wavefront and there are no other active pixels present within the mapping field in the preceding frame (*new wavefront following electrical quiescence in mapping field*). See *Figure 1* for examples.

To identify new wavefronts, each of the active pixels in a given frame was checked for the existence of active pixels in a neighbourhood threshold of the pixel in the preceding frame (implemented using a convolution operator). Pixels without a neighbouring active pixel in the previous frame were defined as new wavefront pixels; otherwise pixels were identified as belonging to a propagated wavefront.

### Histomorphometry and immunohistochemistry

After optical mapping experiments, each left atrial preparation was divided into nine regions (superior septum, mid-septum, inferior septum, LA roof, posterior LA, LA floor, superior lateral LA, lateral LA, and inferolateral LA), and fresh-frozen for subsequent histomorphometry and immunohistochemistry analysis.

Cryostat sections (10 µm) were immunolabelled for connexin43 (Cx43) as previously described.^18^ Sections were incubated with a mouse anti-Cx43 primary antibody (Chemicon MAB 3067) then with a Cy3-conjugated anti-mouse secondary antibody (Jackson). Connexin40 immunolabelling was performed using a goat anti-Cx40 primary antibody (Santa Cruz SC-20466) then with an Alexa Fluor 488-conjugated anti-goat secondary antibody (Invitrogen). The heterogeneity of connexin 40 distribution, the colocalization of connexin 40 and 43 at the intercalated disks, the size of en-face intercalated disks and the proportional occupation by connexin43 of these en-face disks were quantified.

Myocardial fibrosis was assessed by detecting autofluorescence at a wavelength of 488 nm.^19^ The area of fibrosis was quantified using Fiji.^20^

### Computational model

Simulations were run on a canine left atrial surface mesh, adapted from Vigmond *et al.*,^21^ with two-dimensional endocardial and epicardial layers discretely connected using line elements. The Ramirez–Courtemanche–Nattel canine atrial ionic model^22^ was used, with the Kneller *et al.*^23^ formulation for I_KACh_ to incorporate the effects of acetylcholine. Fibre directions were included in the model using a mapping from a human bilayer mesh,^24^ and repolarization heterogeneity was included as differences in cellular ionic conductivities between the LA body, the appendage, and the pulmonary veins (PVs).^25^ The monodomain formulation was solved with the CARPentry simulator (available at https://carp.medunigraz.at/carputils/).

**Figure 2:**
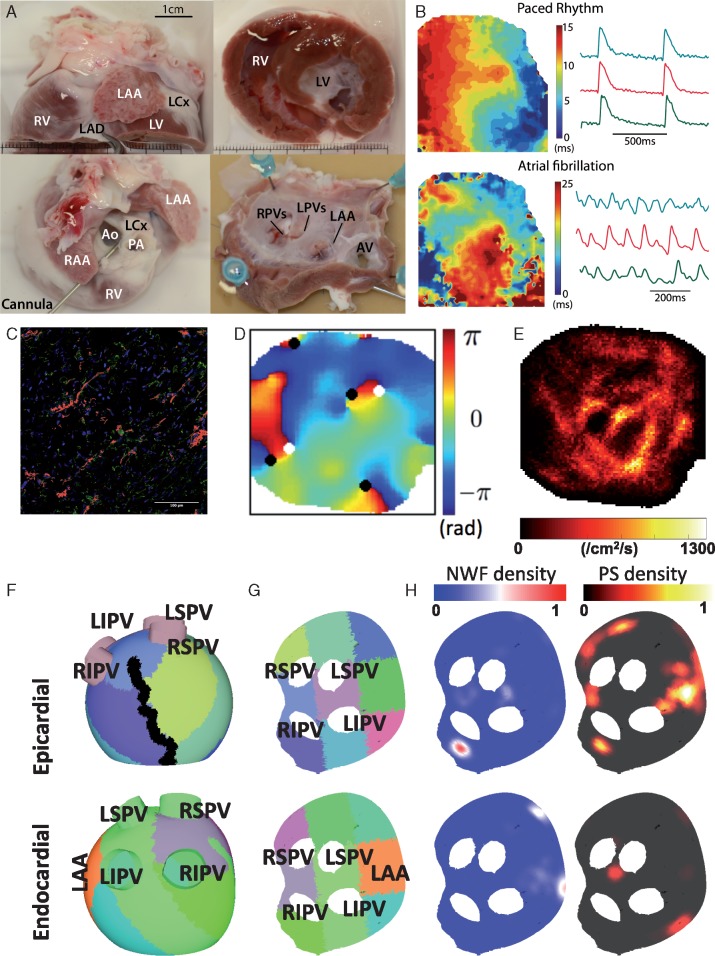
Experimental and computational model set-up. (*A*) Experimental preparation—canine hearts donated by collaborators were dissected and the isolated left atrium perfused via the circumflex artery for left atrial endocardial optical mapping. (*B*) Examples of activation maps and optical action potentials during pacing and in AF for this experimental set-up, generated using the Rhythm GUI.^13^ (*C*) Areas of fibrosis quantified using autofluorescence (red). (*D*) Phase map showing phase singularities (black and white circles, depending on chirality). (*E*) PS density map. (*F*) Computational mesh used for simulations divided into nine regions to correspond with experimental sections used for histomorphometry and immunohistochemistry. An ablation line corresponding to the location used to open the tissue for endocardial optical mapping is also marked in black. The LAA location is also marked for comparison with *B*. (*G*) The mesh is flattened for visualization purposes, in the same way as the experimental preparation. (*H*) Example NWF density and PS density maps. AF, atrial fibrillation; Ao, aorta; AV, aortic valve; LAA, left atrial appendage; LAD, left anterior descending artery; LCX, left circumflex artery; LIPV, left inferior PV; LV, left ventricle; LPVs, left pulmonary veins; LSPV, left superior PV; PA, pulmonary artery; PS, phase singularity; PV, pulmonary vein; RAA, right atrial appendage; RV, right ventricle; RIPV, right inferior PV; RPVs, right pulmonary veins; RSPV, right superior pulmonary vein; NWF, new wavefront.

To reproduce the experimental preparation in which the left atrium was opened to perform endocardial optical mapping, equivalent ablation lines were added in the model. These were along the inter-atrial septum, as shown in *Figure 2F* and were set to have a conductivity of effectively 0 (0.001, to avoid numerical issues). For visualization purposes, each of the endocardial and epicardial layers of the model were flattened to two dimensions (see *Figure 2G*), by removing mesh elements within the ablation lines, and then using a mapping technique that best preserves geodesic distances.^26^

The tissue and cell level properties of the simulated mesh were tuned to one of the experimental preparations (LA1), with different properties in each of the nine regions assessed using the histomorphometry and immunohistochemistry analysis as follows. *I*_K1_ conductance and tissue level conductivity were tuned to the average APD and CV values at baseline pacing for each of the nine regions. Interstitial fibrosis was included in the mesh as microstructural discontinuities, acting as conduction barriers. Mesh edges were stochastically selected as fibrotic with probability depending on both the edge direction compared with the fibre direction (with edges in the longitudinal direction four times more likely to be selected) and the percentage fibrosis measured in the region.^27^

To investigate potential mechanisms underlying new wavefronts, meshes were constructed with different permutations of the following factors: fibrosis on the epicardium only,^28^ or on both endocardial and epicardial surfaces; islands of acetylcholine release on the endocardial surface, on both surfaces, or on neither surface; no gradient in effective refractory period (ERP) between the surfaces, or a shorter ERP on the epicardium^29^; varied connection between the endocardial and epicardial surfaces, ranging from a single connection site, to fully connected. The amount of fibrosis varied between regions in the range 0.2–16.6%, which corresponds to the percentage of model edges that were selected as fibrotic. Islands of acetylcholine release were included as 10 islands per surface, each of radius 2.4 mm, in a random arrangement, following Vigmond *et al*.^21^ Epicardial ERP varied between the nine atrial regions in the range 28.0–35.0 ms. For cases with a difference in ERP between the endocardium and epicardium, the endocardium was modelled with an ERP of 40.0 ms.

## Results

Four experimental preparations were assessed, labelled here as LA1 (left atrial preparation number one), to LA4. For each of the preparations, between 8 and 14 ten-second recordings were taken and analysed offline.

### Dominant frequency and phase singularity locations

**Figure 3:**
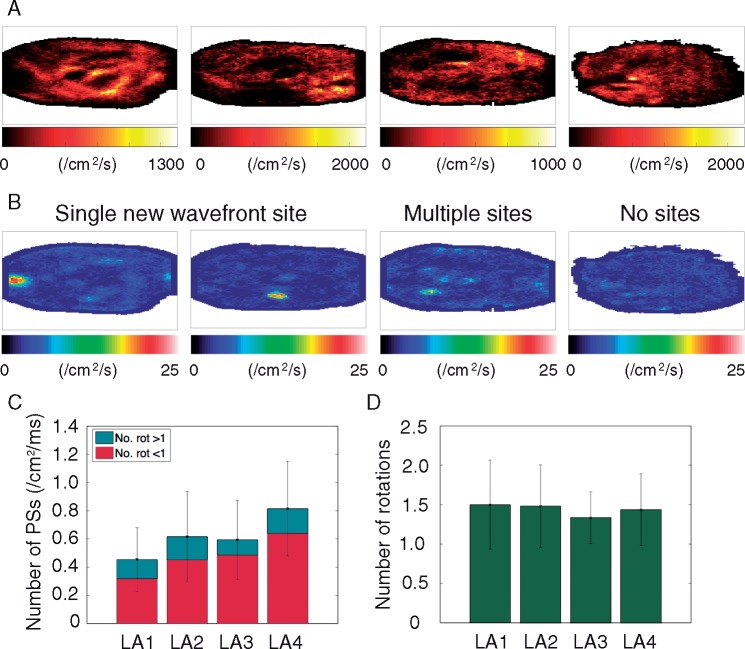
Three out of four of the preparations exhibit clear sites of new wavefront initiation. (*A*) PS density maps. (*B*) Two of the preparations exhibit a single high-density new wavefront initiation site (LA1 and LA2); one preparation shows multiple preferential sites (LA3); and one preparation demonstrates no preferential sites (LA4). (*C*) Average number of phase singularities and rotors vary across preparations and are highest for LA4. (*D*) Average number of rotations are similar across preparations. PS, phase singularity; Rot, rotations.

Mean DF, which varies between the preparations from 14.5 Hz to 33.8 Hz, exhibits a degree of temporal stability. LA1–LA3 have a low PS density on the septal aspect of the left atrium (*Figure 3A*). However, the preferential areas with highest PS density vary across preparations between the lateral regions and the posterior regions. Areas of high PS density are often located around the LA/PV junction. Phase singularity density maps are also stable over time (correlations between recording files: LA1: 0.76 ± 0.11, LA2: 0.63 ± 0.15, LA3: 0.69 ± 0.13, and LA4: 0.70 ± 0.14). Phase singularity initiation locations exhibit greater temporal stability than PS trajectory distribution maps. There is more variability between the PS annihilation location densities.

### New wavefront locations

Three out of four preparations have clear areas with a high-density of new wavefront initiation sites (*Figure 3B*). Two of the preparations (LA1 and LA2) exhibit a single preferential site, whilst one of the preparations (LA3) exhibits multiple sites of high-density. The final preparation (LA4) did not have any clear preferential regions. These findings are reproduced across sequential recordings, showing there is stability over time.

### Correlation between frequency, phase singularity distribution maps and new wavefront sites

The linear correlation between DF, PS distribution maps and new wavefront location maps were calculated for each of the recordings and the mean values for each of the hearts were low (<0.11 for DF and PS distribution maps; <0.13 for PS distribution maps and new wavefront locations; and <0.25 for DF and new wavefront locations).

### Phase singularity number and number of rotations

The overall mean number of PSs per cm^2^ per ms across all of the recordings were calculated for each of the preparations and are shown as those that last less than a rotation and those that last for more than a rotation in *Figure 3C*, where the shorter lasting PSs represent short-lived wavefronts.

The preparation that did not have any preferential sites of new wavefront initiations (LA4) has a greater number of PSs per unit area measured on the endocardium than the other three preparations (*Figure 3C*, LA4 0.81 ± 0.34 vs. LA1 0.45 ± 0.23, LA2 0.61 ± 0.28, LA3 0.59 ± 0.28 PS cm^2^/ms). This suggests that the nature of AF may exhibit differences between the preparations.

### Endocardial rotational activity vs. new endocardial wavefronts

The differences between the preparations in terms of the number of PSs and the number of new wavefront initiations were determined in order to investigate the mechanisms for AF, to discern whether rotors, multiple wavelets or focal wavefronts maintain the activity on the endocardium.

There was a single dominant mechanism for sustaining AF in each of the LA preparations. In preparations with low numbers of PSs, we observed stable and clear preferential sites for new wavefront initiations (e.g. LA1). Conversely, in preparations without clear preferential sites of new wavefront initiations, there were higher numbers of active tissue pixels and PSs.

**Figure 4:**
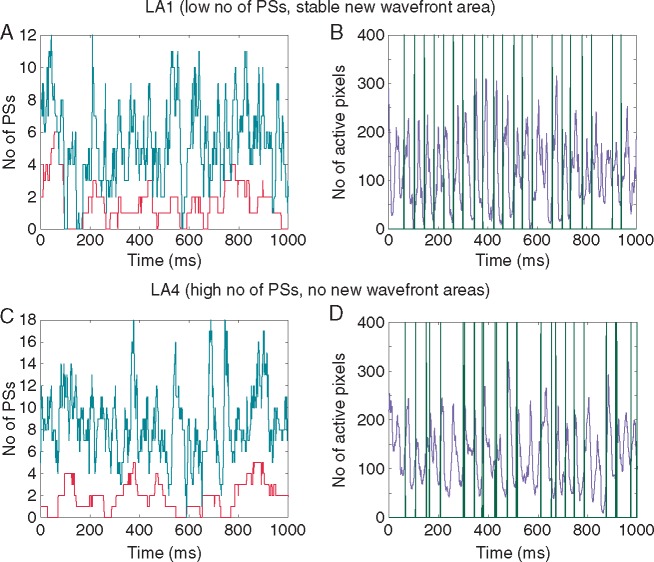
Investigating the number of rotors, PSs, active tissue pixels and new wavefronts over time indicates the nature of AF. For LA1, AF is sustained by new wavefronts (*A* and *B*). For LA4, AF is sustained by rotational activity (*C* and *D*). (*A*) Number of rotors (PS lasting greater than one rotation, in red) and number of PSs (teal line) over time; (*B*) Number of active pixels in violet and timings of new wavefront initiations in green. The timings of the new wavefront initiations can be seen to coincide with times when the number of active tissue pixels drops to zero, triggering the re-initiation of AF in the mapping field. (*C*) Number of rotors (PS lasting greater than one rotation, in red) and number of PSs (teal line) over time. (*D*) Number of active pixels in violet and timings of new wavefront initiations in green, where the new wavefront initiations can occur anywhere in the domain. In this preparation, new wavefront initiations do not appear to play a dominant role in the wavefront patterns observed on the endocardium. AF, atrial fibrillation; PS, phase singularity.

*Figure 4* demonstrates a technique for investigating the mechanisms sustaining AF. *Figure 4A* shows the number of PSs and the number of PSs that last greater than one rotation (rotors). There are instances with a large number of rotors in which the activity is maintained by these electrical rotors. However, there are also instances when the number of rotors drops down to zero. *Figure 4B* shows the number of active pixels on the endocardium over time. There are instances when there are no more activation wavefronts within the mapped endocardial region and this score drops to zero. In this instance, AF would terminate unless a wavefront enters the mapping field from the surrounding tissue, or there is a new focal beat on the mapped surface in the tissue. In *Figure 4B*, the activation times from the new wavefront area are plotted alongside the number of active pixels in the tissue, and it can be seen that increases in activity from the state where there are no activation wavefronts (electrical quiescence) can be attributed to the new wavefronts from this area. Electrical activity in the mapping field appears to restart due to new wavefront initiations from the identified preferential site.

To determine whether the new wavefronts in the preferential sites were responsible for restarting the electrical activity following electrical quiescence in the mapping field, the origin of the electrical activity was assessed. In two of the preparations (LA1 and LA2), the density of new wavefronts following electrical quiescence was significantly higher in the preferential region than in the rest of the tissue, indicating that the high-density site played a relevant role in both preparations.

In contrast, LA4 did not have any clear preferential sites of new wavefront initiations. In this case, the number of active tissue pixels tended to be higher (i.e. a lower frequency of electrical quiescence, see *Figure 4C*). Activation times for all new wavefront initiations, regardless of location, were plotted alongside the number of active pixels in *Figure 4D*. For this preparation, new wavefronts do not play a dominant role in the activity observed on the endocardial surface, and there is a higher density of endocardial PSs than for the other preparations.

### Comparing new wavefront and rotor cycle lengths

For one of the preparations with one preferential site of new wavefront initiations (LA2), the high-density site was found to have an equal median CL to the median rotor CL (78 ms and 77 ms; *P* = 0.73, by Mood's median test, a non-parametric test for determining whether medians of two datasets are equal). For the other preparation with one preferential site of new wavefront initiations (LA1), the median CLs were unequal, but of similar magnitude (new wavefront CL 45 ms; rotor CL 38 ms; *P* < 0.005, Mood's median test).

**Figure 5:**
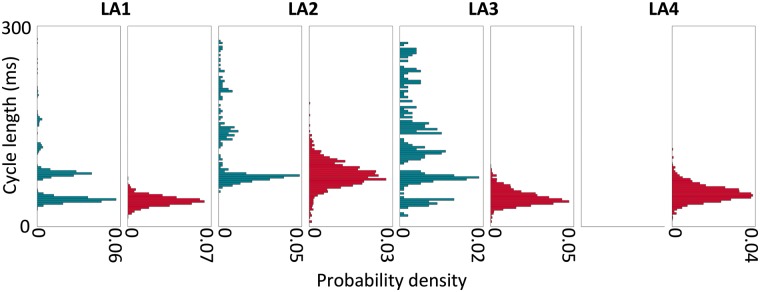
Probability density histograms show distributions of CLs for the high-density site of new wavefronts and for rotors exhibit differences between preparations. CLs measured during the recordings are displayed as probability density histograms, with teal histograms showing new wavefront CLs and red histograms showing rotor CLs. For LA4, there were no preferential sites of new wavefront initiations, so only the distribution of rotor CLs is considered. CL, cycle length.

For the preparation with multiple sites (LA3), the largest site by area was found to have a higher median CL than the median rotor CL (110 ms vs. 41 ms; *P* < 0.005, Mood's median test). This agrees with the observation that the largest site did not play a dominant role in maintaining the arrhythmia following electrical quiescence. *Figure 5* shows the distribution of new wavefront and rotor CLs for each of LA1–LA4, displayed as probability density histograms. For each of LA1–LA3, the new wavefront area CL was observed to cluster to some degree at harmonics, representing instances when the tissue was refractory to the first attempted breakthroughs. For LA3, although the overall median new wavefront CL is higher than the median rotor CL, there is a cluster of new wavefront CLs with median close to the median rotor CL.

The rotor CLs varied between preparations in line with the DF variation.

### No correlation between sites of phase singularities and new wavefronts with connexin/fibrosis content

**Figure 6:**
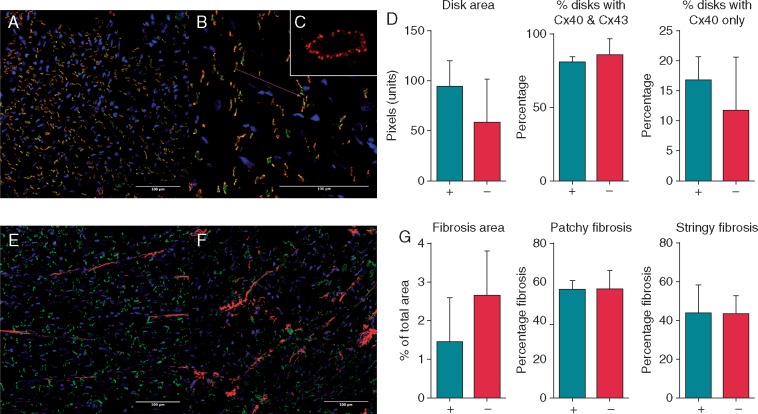
No correlation between phase singularity location and connexin or fibrosis content. (*A* and *B*) Connexin40 (green) and connexin43 (red) immunolabel viewed at ×20 and ×40 magnification, respectively. Areas of Cx40 and Cx43 colocalization shown in yellow. (*C*) Intercalated disk viewed en face at ×60 magnification for disk size quantification. (*D*) Comparison of regions with high rotor densities (+) vs. regions with low rotor densities (−) showed no differences in intercalated disk size or Cx40 or Cx43 distribution/percentage. (*E* and *F*) Areas of fibrosis quantified using autofluorescence (red). (*G*) Percentage of fibrosis by area was compared between left atrial regions with high rotor densities (+) vs. regions with low rotor densities (×). Fibrosis area was not different between these regions. Fibrosis was subdivided into stringy (interstitial fibrosis) (shown in *E*) and patchy fibrosis (*F*), which was also not different between groups.

We investigated the possible structural determinants of the locations of new wavefronts and preferential sites of phase singularities. The connexin content/distribution and the fibrosis content at the sites of new wavefronts and of phase singularities were not different to the connexin/fibrosis content at other sites (see *Figure 6*). Specifically, *Figure 6D* shows that intercalated disk size, connexin40 percentage and connexin43 percentage did not vary between high and low PS density regions. *Figure 6G* shows that total fibrosis area, interstitial fibrosis area and patchy fibrosis area are not different between high and low PS density regions.

### Simulations

To investigate the potential causes of stable repetitive new wavefront sites, computational simulations were run with different distributions of fibrosis, acetylcholine islands, gradients of ERP between the surfaces, and varied numbers of connections between endocardial and epicardial surfaces. Simulations exhibited a range of activities, including dissociation and breakthrough between the endocardial and epicardial surfaces, as well as rotational activity anchored to areas of fibrosis, long APD, or around the LA/PV junction.

**Figure 7:**
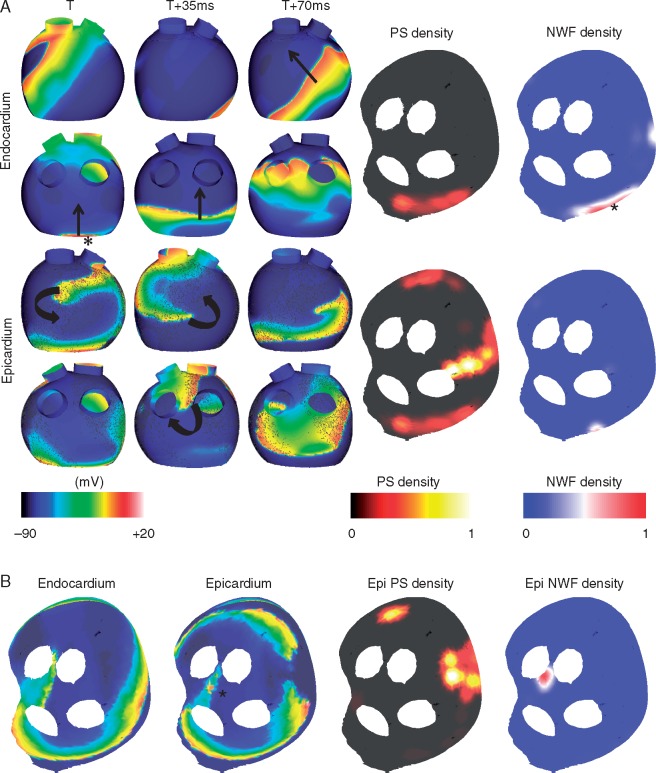
Simulations demonstrate preferential sites of new wavefront breakthrough, together with rotational activity anchored to fibrosis and long APD, or meandering around the LA/PV junction. (*A*) A site of wavefront breakthrough can be seen on the posterior wall of the endocardium, by the mitral valve (indicated by the asterisk). Transmembrane plots are shown in anteroposterior (top) and posteroanterior view (bottom), for the endocardium and epicardium. PS density and NWF density maps are shown in the flattened view for the endocardium and epicardium (see *Figure 2G* for orientation). The asterisk shows the site of breakthrough. High PS density is seen on the epicardium, in an area of increased fibrosis and long APD. (*B*) This simulation exhibits epicardial breakthrough (asterisk), and endocardial reentry around the LA/PV junction. No breakthrough is observed on the endocardium so PS density and NWF density maps are shown for the epicardium only. A single NWF site is seen on the epicardium. APD, action potential duration; LA, left atrium; PV, pulmonary vein; PS, phase singularity; NWF, new wavefront.

*Figure 7* shows two example set-ups with four connection sites, in which a single site of high new wavefront density is seen. *Figure 7A* shows results from a simulation with epicardial fibrosis; acetylcholine islands, with a higher concentration on the endocardium; and with no gradient in APD between the endocardium and epicardium. A single site of wavefront breakthrough can be seen on the LA posterior wall, close to the mitral valve (indicated by the asterisk). Activity on the epicardial wall is driven by rotational activity that meanders around an area of high fibrosis density and long APD on the anterior wall, and a second rotor on the posterior wall that meanders around the left inferior PV/LA junction. The PS density maps indicate a higher density on the epicardium in the area of fibrosis and long APD, and the new wavefront density maps indicate a high-density area on the endocardium (indicated by the asterisk).

*Figure 7B* shows another example with four connection points, with only a single new wavefront location. For this simulation, acetylcholine effects are homogeneous and the only difference between the endocardial and epicardial surfaces is the presence of epicardial fibrosis. This fibrosis causes the epicardial wavefronts to break up and results in a less stable activity, while the endocardial activity anchors around the LA/PV junction. The asterisk, which is at a site between the superior veins, indicates breakthrough.

**Figure 8:**
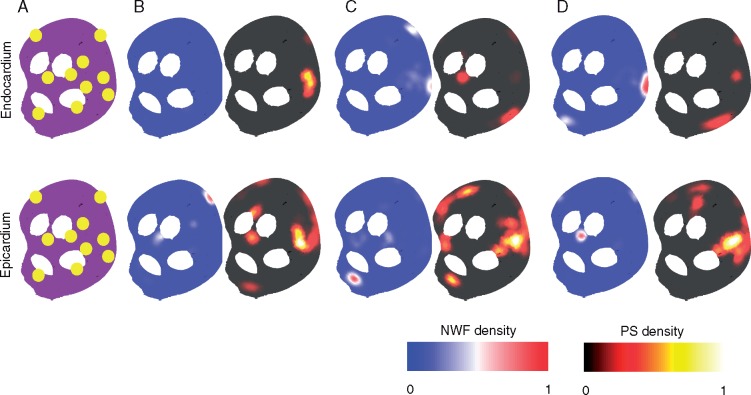
A small number of preferential sites for new wavefronts are observed in simulations with a larger number of connection points. (*A*) Ten randomly generated connection locations shown in yellow. (*B*) Set-up with epicardial fibrosis, identical acetylcholine islands, and an APD gradient between surfaces. The APD gradient between the surfaces means that no new wavefronts are seen on the endocardial surface. A single preferential site of new wavefronts can be seen on the epicardium. (*C*) Set-up with epicardial fibrosis, identical acetylcholine islands, and no APD gradient between surfaces. The removal of the APD gradient means breakthrough occurs on both surfaces. There is more complicated conduction on the epicardium than endocardium due to the epicardial fibrosis, leading to more areas of high PS density. (*D*) Set-up with epicardial fibrosis, identical acetylcholine islands with increased endocardial acetylcholine concentration, and no APD gradient between surfaces. PS density maps are similar to *C*, but preferential sites of breakthrough are in different locations. These sites are different for the endocardium and epicardium. APD, action potential duration; PS, phase singularity; NWF, new wavefront.

*Figure 8* demonstrates that there are small numbers of new wavefront sites for simulations with larger numbers of connection locations. The ten connection locations for these simulations are shown in *Figure 8A* (in yellow). These simulations have identical epicardial fibrosis, but a difference in either acetylcholine concentration between the surfaces, or in the epicardial-endocardial APD gradient. This leads to similar PS density maps across the different simulations (compare B–D for corresponding endocardial or epicardial maps), but different preferential sites for new wavefront locations.

## Discussion

### Summary of findings

We developed a combined experimental and modelling framework to identify the mechanisms sustaining AF, including rotational wavefronts and sites of repetitive new wavefronts. There was a single dominant mechanism in each of the preparations. In the cholinergic AF preparations, AF was sustained by either endocardial rotational activity or by new wavefront initiations. Preferential PS sites existed with low PS density on the septal region in three of the four preparations, and high PS density around the LA/PV junction. Computational simulations demonstrated a small number of preferential new wavefront locations determined by the interplay between fibrosis distribution, acetylcholine concentration and heterogeneity in repolarization. The methodology presented here may be used to identify the mechanisms sustaining AF, and to assess the suitability of treatment strategies.

### Repetitive endocardial wavefronts to sustain atrial fibrillation

A novel method was developed in this study to identify new wavefronts from phase. This has the great advantage that it does not require any activation time assignment. Clear patterns are seen in many of the new wavefront spatial distribution maps, demonstrating efficacy of the technique.

Focal sources have also been identified in many other studies.^4^^,^^7^^,^^30^ New wavefronts due to endocardial-epicardial dissociation have been observed, although in contrast to the findings here, these were not in repeated locations.^8^ There are some studies with new wavefronts occurring in repeated locations, including the study of Filgueiras-Rama *et al.*^31^ in which organized focal wavefronts were detected at the same site and with the same direction over four or more sequential beats. Ikeda *et al.*^32^ also identified areas of new wavefronts in canine cholinergic AF, but in the RA. These wavefronts coexisted with meandering rotational waves, but were not present in the case of anchored re-entry.

### The origin of new wavefronts: triggered, transmural conduction, or rotors?

New wavefront initiation sites were found to be functionally important in sustaining AF in the endocardial mapping field in three of the four preparations investigated here. However, the source of these new wavefronts are unknown. In particular, it is unclear whether they were due to triggered activity, or transmural conduction. There is evidence to suggest that ACh may induce ectopy in the PVs and appendage,^33^ but it is thought to suppress triggered activity outside of these areas.^10^^,^^34^ Thus, it is unlikely that new wavefronts are due to a triggered source with fixed CL. The Fedorov laboratory has recently demonstrated that intramural fibrotic strands, together with fibre misalignment and variations in atrial wall thickness may create microanatomic tracks for stable re-entrant AF drivers.^35^^,^^36^

### Bilayer model simulations exhibit a small number of preferential sites for new wavefronts

Our computational simulations offer insight into why a small number of new wavefront locations may exist in substrates with a large number of connections between the endocardial and epicardial surfaces. *Figure 7A* shows an example with a single endocardial site of new wavefronts, similar to LA1 and LA2. *Figure 7B* has no endocardial sites of new wavefronts, similar to LA4. New wavefront locations in the computational model depend on the interplay between repolarization gradients and fibrosis distribution. Simulations with a shorter APD on the epicardium exhibit sites of new wavefronts on the epicardium, but not the endocardium (e.g. *Figure 7B*). Rotational activity may anchor to areas of fibrosis, long APD or around the LA/PV junction (e.g. *Figure 7A*). *Figure 8* demonstrates that simulations with a large number of connection sites may exhibit only a small number of new wavefront locations, explaining this occurrence in the experimental preparation. New wavefront locations vary between simulations, despite similar PS distributions. Our findings imply that electrical measurements should be interpreted differently according to whether they are recorded on the endocardial or epicardial surface, and the transmural aspect of AF should be considered when planning ablation treatment approaches.

Gharaviri *et al.*^37^ also simulated two surfaces with varied connections between the endocardium and epicardium. They simulated a large range of number of connections to find that endocardial–epicardial dissociation and AF duration both increase as the number of connections in their model is decreased. We investigated a smaller range for the number of connections, but also included heterogeneity in repolarization and fibrosis in our simulations. Our findings agree in that we also observe endocardial–epicardial dissociation for 6–10 connections, and we offer an extension to investigate the reasons behind preferential new wavefront locations.

### Endocardial rotational activity vs. new endocardial wavefronts

The developed techniques elucidate wavefront dynamics in the preparation studied here, demonstrating the interplay between endocardial rotational activity and new endocardial wavefronts. In particular, by considering the number of PSs that last for greater than one rotation, the total number of PSs and the new wavefront activation times, it is possible to discern whether the activity is maintained within the mapping field by rotors, multiple wavefronts or focal sources, respectively. By assessing the frequencies of arrhythmia termination, new wavefronts from the high-density regions and new wavefronts following electrical quiescence in the mapping field, the mechanisms sustaining AF can be determined, and foci responsible for near-instantaneous reinitiation of AF can be identified. This suggests that the techniques developed in our study can be used to investigate arrhythmia dynamics. Identification of foci responsible for sustaining AF may have clinical utility as potential ablation targets.

### Dominant frequency and phase singularity stability

Dominant frequency was found to be relatively stable for each preparation over the AF duration. There is conflicting evidence in the literature on the stability of DF during AF; Sanders *et al.*^5^ found DF was stable in human AF, whilst Salinet *et al.*^6^ found frequency to be temporally unstable.

All preparations exhibited preferential regions for PS location and a degree of stability in distribution over time. For three of the preparations (LA1, LA2, and LA3), the PS density is low in the septal region of the tissue. Similar to our findings, some studies have suggested that AF driver locations show a degree of spatio-temporal stability on a regional basis.^4^^,^^7^

### Correlation between frequency, phase singularity distribution maps and new wavefront initiation distribution maps

There was very little correlation between DF, PS distribution maps and new wavefront sites in the preparations studied here. Drivers, whether focal or reentrant, are typically thought to coincide with areas of high-frequency.^38^^,^^39^ PS distribution maps with a different threshold for PS inclusion (for example, requiring PSs to undergo a full rotation) may show an improved correlation; however, the change is expected to be minimal as PS maps with thresholds of half a rotation and a full rotation agreed in initial parameter testing. Equally applying a spatial filter to the maps before calculating the correlation may improve the correlation.

### Limitations

A key limitation of the work presented here is the inability to discern the cause of new wavefronts from the optical mapping data alone. Mapping of both the endocardial and epicardial surface using optical mapping or electrode arrays would help with the interpretation of the origin of new wavefronts. For example, electrogram morphology could indicate the source of the waves as triggered or breakthrough. The application of ion channel blockers may also aid with determining the origin of focal wavefronts. Our preliminary parameter testing demonstrated that the bin size used to spatially smooth the voltage and phase data affected the number of short-lived PSs, but did not have a large effect on the number of long lasting PSs.^16^ As such, the spatial and temporal resolution (pixel size of 0.44 mm × 0.44 mm, at 1 ms) used here is likely to be sufficient to detect rotors and new wavefronts. However, it is possible that measurements at the single cell level may offer additional insight into the mechanisms underlying re-entry; for example, our recent study demonstrated re-entry cores composed of conduction block lines.^40^ However, this resolution is not attainable in tissue. Our simulations produce the range of behaviours typical of the experimental set-up. However, the model does not replicate the exact arrhythmia dynamics observed experimentally because model tuning was based on average APD and CV values, considered over nine atrial regions, and we used a bilayer rather than volumetric model.

## Conclusions

Our novel combined experimental and modelling framework identified the dominant mechanisms for sustaining AF in canine cholinergic AF, including rotational wavefronts and sites of repetitive new wavefronts. There was a single dominant mechanism (rotors, multiple wavelets, or new wavefronts) for sustaining AF in each of the preparations. Preferential PS sites exist with low PS density on the septal region of all preparations, and high PS density around the LA/PV junction. Computational simulations demonstrated that despite numerous connections between the atrial layers, only a small number of connections are functionally important in sustaining AF, with preferential new wavefront locations determined by the interplay between fibrosis distribution, acetylcholine concentration and heterogeneity in repolarization.
